# Synovial fluid biomarkers associated with osteoarthritis severity reflect macrophage and neutrophil related inflammation

**DOI:** 10.1186/s13075-019-1923-x

**Published:** 2019-06-13

**Authors:** Collin A. Haraden, Janet L. Huebner, Ming-Feng Hsueh, Yi-Ju Li, Virginia Byers Kraus

**Affiliations:** 1 Duke Molecular Physiology Institute, Duke University School of Medicine, Box 104775, 300 North Duke St, Durham, NC 27701 USA; 2 Department of Biostatistics and Bioinformatics, Duke University School of Medicine, Durham, NC USA; 3 Department of Medicine, Duke University School of Medicine, Durham, NC USA

**Keywords:** Osteoarthritis, Joint pain, Macrophage, Neutrophil, Inflammation, Biomarker, Knee, Severity, Radiograph

## Abstract

**Background:**

To identify a synovial fluid (SF) biomarker profile characteristic of individuals with an inflammatory osteoarthritis (OA) endotype.

**Methods:**

A total of 48 knees (of 25 participants) were characterized for an extensive array of SF biomarkers quantified by Rules Based Medicine using the high-sensitivity multiplex immunoassay, Myriad Human InflammationMAP® 1.0, which included 47 different cytokines, chemokines, and growth factors related to inflammation. Multivariable regression with generalized estimating equations (GEE) and false discovery rate (FDR) correction was used to assess associations of SF RBM biomarkers with etarfolatide imaging scores reflecting synovial inflammation; radiographic knee OA severity (based on Kellgren-Lawrence (KL) grade, joint space narrowing, and osteophyte scores); knee joint symptoms; and SF biomarkers associated with activated macrophages and knee OA progression including CD14 and CD163 (shed by activated macrophages) and elastase (shed by activated neutrophils).

**Results:**

Significant associations of SF biomarkers meeting FDR < 0.05 included soluble (s)VCAM-1 and MMP-3 with synovial inflammation (FDR-adjusted *p* = 0.025 and 1.06 × 10^−7^); sVCAM-1, sICAM-1, TIMP-1, and VEGF with radiographic OA severity (*p* = 1.85 × 10^−5^ to 3.97 × 10^−4^); and VEGF, MMP-3, TIMP-1, sICAM-1, sVCAM-1, and MCP-1 with OA symptoms (*p* = 2.72 × 10^−5^ to 0.050). All these SF biomarkers were highly correlated with macrophage markers CD163 and CD14 in SF (*r* = 0.43 to 0.90, FDR < 0.05); all but MCP-1 were also highly correlated with neutrophil elastase in SF (*r* = 0.62 to 0.89, FDR < 0.05).

**Conclusions:**

A subset of six SF biomarkers was related to synovial inflammation in OA, as well as radiographic and symptom severity. These six OA-related SF biomarkers were specifically linked to indicators of activated macrophages and neutrophils. These results attest to an inflammatory OA endotype that may serve as the basis for therapeutic targeting of a subset of individuals at high risk for knee OA progression.

**Trial registration:**

Written informed consent was received from participants prior to inclusion in the study; the study was registered at ClinicalTrials.gov (NCT01237405) on November 9, 2010, prior to enrollment of the first participant.

**Electronic supplementary material:**

The online version of this article (10.1186/s13075-019-1923-x) contains supplementary material, which is available to authorized users.

## Background

Although the role of inflammation in osteoarthritis (OA) has been heavily debated, cumulative evidence from ultrasound and magnetic resonance imaging (MRI) demonstrates inflammation in the majority of individuals with radiographic knee OA [1–4]. Moreover, inflammation in the form of MRI effusion or Hoffa’s fat pad synovitis predict increased risk of incident radiographic knee OA [5, 6]. Using 99mTc-etarfolatide nuclear imaging, we have detected a high frequency of folate receptor-positive cells (FR+), representing activated macrophages [7] and neutrophils [8], in knee OA.

Both macrophages and neutrophils respond to and perpetuate local inflammation. In response to damage-associated molecule patterns (DAMPs) and inflammatory cytokines, macrophages can become activated in the joint organ and modulate the local cytokine, growth factor, and matrix metalloproteinase (MMP) environment [9, 10]. Neutrophils secrete a number of proteinases including elastase [11] capable of degrading many targets including elastin, collagens, and proteoglycans [12]. In addition, neutrophil elastase can upregulate the expression of proteinase-activated receptors (PARs) and activate them though cleavage [13]. When activated, PARs perpetuate synovitis and cartilage loss and cause hyperalgesia and osteophyte formation [14, 15].

We recently discovered that synovial fluid (SF) biomarkers CD14 and CD163, soluble forms of macrophage cell surface markers, and SF elastase, secreted by neutrophils [8, 16], function as quantitative traits of the etarfolatide-positive phenotype and predict knee OA progression over 3 years [8, 16]. Using the SF and plasma samples from individuals with knee OA, our goal in this substudy was to investigate the association of an extensive array of SF biomarkers with knee OA inflammation based on etarfolatide phenotyping, radiographic manifestations of knee OA, and OA symptoms. Patients were recruited for the original study on evidence of radiographic OA and symptoms and not inflammatory OA clinical signs. Thus, this substudy was designed to evaluate inflammation in “typical” OA joints. We hypothesized that the synovial fluid biomarkers associated with etarfolatide imaging and knee radiographic OA severity would provide a molecular biological profile for identifying the subset of subjects with an inflammatory OA endotype [17].

## Patients and methods

### Study description

This is a novel substudy of an investigator-initiated single-center clinical trial conducted at Duke University Medical Center performed as previously described [7] and with authorization from the Food and Drug Administration (IND 108,677). The study was registered at ClinicalTrials.gov (NCT01237405) prior to enrollment of the first participant. Participants ≥18 years old were recruited on the basis of radiographic OA (Kellgren-Lawrence [18] grade 1–4 severity) in at least one knee and knee pain in the index knee on most days of any 1 month in the last year [19]. A total of 25 participants were enrolled. Exclusion criteria were as previously described [7]. This clinical investigation was conducted according to the Declaration of Helsinki principles, with the approval of the Duke University Medical Center Institutional Review Board. Written informed consent was received from participants prior to inclusion in the study.

### Imaging

Knee radiographs (*n* = 50) of both knees of 25 patients were obtained as previously described using an optimal and standardized method with the SynaFlexer™ frame [20]. Radiographs were graded blinded to other imaging and clinical data by the consensus of two experienced readers with high inter-rater reliability as previously described [21]. Radiographs were scored for Kellgren-Lawrence (KL) [18] grade (0–4), joint space narrowing (JSN, 0–3), and osteophyte severity (OST, 0–3) using a standard atlas [22]. All 25 individuals also underwent Etarfolatide imaging of both knees and the whole body as previously described with high intra-rater reliability of scoring (*κ* = 0.68–0.90) [7]. For these analyses, severity of synovitis due to the presence of cells positive for folate receptor, reflecting both activated macrophages and neutrophils, was quantified by summing synovial etarfolatide scores for medial and lateral compartments of the knee.

### Clinical symptoms

All 50 knees were scored semi-quantitatively (normal/none 0, mild 1, moderate 2, or severe 3) for self-reported intensity of joint pain ascertained by the NHANES I criterion [19] (pain, aching or stiffness (PAS) on most days of any 1 month in the last year).

### Biospecimen collection

SF was collected from 48 knees either directly (*n* = 28) or by small volume (10 ml) saline lavage (*n* = 20) when direct aspiration failed. Two patients refused joint aspirations of their second knee. The dilution factor of SF obtained by small volume saline lavage was determined, and corresponding biomarker concentrations were corrected using a previously established urea-based methodology [23]. Whole blood was obtained immediately after SF aspiration using Truculture Null (782–001086, RBM) tubes with subsequent manual separation of plasma supernatants from cells using valve filter Seraplas® V11 (53.677, Sarstedt). All samples were stored at − 80 °C until analysis.

### Biomarker quantification

Biomarkers in SF and plasma were quantified by Rules Based Medicine (RBM; Austin, TX) using the high-sensitivity multiplex immunoassay Myriad Human InflammationMAP® 1.0. Measured biomarkers included 47 different cytokines, chemokines, and growth factors related to inflammation. For purposes of statistical analyses, biomarkers were excluded if > 25% of the samples had out-of-range low values; excluded biomarkers were brain-derived neurotropic factor (BDNF), C-reactive protein (CRP), eotaxin-1, granulocyte macrophage colony-stimulating factor (GM-CSF), interferon gamma (IFN), interleukin-1 beta (IL-1β), interleukin-2 (IL-2), interleukin-3 (IL-3), interleukin-4 (IL-4), interleukin-5 (IL-5), interleukin-6 (IL-6), interleukin-7 (IL-7), interleukin-8 (IL-8), interleukin-10 (IL-10), interleukin-15 (IL-15), interleukin-17 (IL-17), interleukin-18 (IL-18), interleukin-23 (IL-23), interleukin-12 subunit p40 (IL-12p40), interleukin-12 subunit p70 (IL-12p70), macrophage inflammatory protein-1 alpha (MIP-1α), macrophage inflammatory protein-1 beta (MIP-1β), matrix metalloproteinase-2 (MMP-2), T-cell specific protein (RANTES), tumor necrosis factor alpha (TNFα), tumor necrosis factor beta (TNFβ), tumor necrosis factor receptor 1 (TNFR1), and von Willebrand factor (vWF).

A total of 17 SF biomarkers met inclusion criteria for statistical analysis. For retained biomarkers, out-of-range low concentrations were imputed as ½ the lower limit of detection (LLOD; *n* = 34 concentrations representing 4% of total determinations); out-of-range high values were imputed as twice the upper limit of detection (ULOD; *n* = 1 concentration representing 0.1% of total determinations). Intra- and inter-assay coefficients of variance for the retained biomarkers, as determined by RBM, were < 7% and < 15%, respectively (Table 1). The mean and interquartile range of SF and plasma biomarker concentrations and the number outside limits of detection are reported in Table 1.

**Table 1:** Descriptive statistics for included biomarkers

	Synovial fluid				Plasma							
Biomarker	*N*	*N* outside LOD	Mean	IQR	*N*	*N* outside LOD	Mean	IQR	LLOD	Unit	Intra-assay CV (%)	Inter- assay CV (%)
A2M	48	0	0.17	0.11	25	0	0.42	0.11	0.051	mg/mL	7.26	13.75
AAT	48	0	0.53	0.46	25	0	1.01	0.23	0.0065	mg/mL	4.93	9.33
B2M	47	1	1.10	0.90	25	0	0.76	0.27	0.15	μg/mL	3.83	6.33
C3	47	1	0.13	0.14	25	0	0.57	0.18	0.00065	mg/mL	5.87	7.33
Factor7	44	4	66.29	74.91	25	0	484.88	143.00	5.60	ng/mL	3.13	8.00
Fibrinogen	44	4	0.08	0.08	25	0	3.69	1.33	0.0056	mg/mL	5.47	10.67
FRTN	48	0	271.66	229.63	24	1	41.14	41.00	6.23	ng/mL	5.67	11.33
sICAM-1	46	2	21.55	20.52	25	0	55.30	17.50	4.78	ng/mL	3.60	8.33
IL-1ra	44	4	273.29	184.00	25	0	278.08	332.00	250.00	pg/mL	4.87	7.67
MCP-1	46	2	218.52	195.96	25	0	381.98	166.00	9.56	pg/mL	3.53	6.00
MMP-3	45	3	1.16 × 10^6^	5.75 × 10^5^	25	0	1.22 × 10^4^	5.30 × 10^3^	5.07 × 10^3^	pg/mL	3.87	8.67
MMP-9	43	5	30.50	15.1	23	2	15.00	6.50	41.4	ng/mL	4.53	8.33
SCF	45	3	198.56	77.00	25	0	94.75	38.50	195.6	pg/mL	3.47	6.67
TIMP-1	48	0	177.44	201.05	25	0	37.14	16.80	9.575	ng/mL	3.80	10.00
sVCAM-1	45	3	292.10	290.80	25	0	282.36	90.00	4.265	ng/mL	6.60	14.00
VDBP	47	1	65.15	47.27	25	0	126.89	73.70	0.635	μg/mL	3.60	9.67
VEGF	46	2	778.06	873.36	25	0	188.80	45.00	18.14	pg/mL	4.13	8.00

Biomarkers listed yielded measurable values for ≥ 75% of samples

*Abbreviations*: *LOD* limit of detection, *IQR* interquartile range, *LLOD* lower limit of detection, *CV* coefficient of variation, *A2M* alpha-2-macroglobulin, *AAT* alpha-1-antitrypsin, *B2M* beta-2-microglobulin, *C3* complement C3, *FRTN* ferritin, *sICAM-1* soluble intercellular adhesion molecule 1, *IL-1ra* interleukin-1 receptor antagonist, *MCP-1* monocyte chemotactic protein 1, *MMP-3* matrix metalloproteinase-3, *MMP-9* matrix metalloproteinase-9, *SCF* stem cell factor, *TIMP-1* tissue inhibitor of metalloproteinases 1, *sVCAM-1* soluble vascular cell adhesion molecule-1, *VDBP* vitamin D-binding protein, *VEGF* vascular endothelial growth factor

### Statistical analysis

We evaluated the association of SF and plasma biomarker concentrations with synovial inflammation (based on Etarfolatide imaging), radiographic OA severity and OA symptoms. Due to the inclusion of measurements from two knees of each patient, generalized estimating equations (GEE) were used to account for within-subject correlation using exchangeable variance-covariance matrix. The GEE model includes the main predictor of interest (the biomarker), age, gender, and body mass index (BMI) for each outcome of interest. False discovery rate (FDR) was computed using methodology described by Benjamini and Hochberg [24]. Significant results were determined based on an FDR-adjusted *p* value ≤ 0.05. Significant SF RBM biomarkers from these analyses were further evaluated for correlation with SF CD14, SF CD163, and SF neutrophil elastase (measured previously on these samples as described [8, 16]) using the Bland and Altman method (rmcorr package in R) to compute repeated measures correlation coefficients [25]. For the correlation between SF and plasma biomarkers, we averaged SF biomarkers across both knees before computing Spearman correlations. These GEE, Bland and Altman correlations, and Spearman analyses were performed using R (https://www.r-project.org/). To determine if SF biomarker concentrations were statistically significantly higher than plasma (and therefore potentially of joint tissue origin), the plasma concentrations and the average SF biomarker concentration of both knees were evaluated with the one-sided Wilcoxon signed-rank test using JMP® Pro, Version 13 (SAS Institute Inc., Cary, NC). A protein functional association network plot was generated using STRING v10.5 database (http://string-db.org) [26].

## Results

### Etarfolatide scan cohort

Participants (*n* = 25) were of mean age 62.4 ± 15.8 years (range 30–89), of mean BMI 29.2 ± 4.8 kg/m2 (range 22.5–38.4), and 72% female. The majority (76%) of participants had moderate to severe bilateral radiographic knee OA with 24% KL grade 1, 58% KL grade 2–3, and 18% KL grade 4.

### Association of synovial fluid biomarkers with severity of OA inflammation, structural features, and symptoms

Of the 17 biomarkers included for statistical analyses, MMP-3 and sVCAM-1 were associated with knee inflammation as assessed by etarfolatide imaging (FDR < 0.05); sICAM-1 and TIMP-1 were marginally associated with inflammation (FDR = 0.090, 0.097). Five biomarkers (MMP-3, sVCAM-1, sICAM-1, TIMP-1, and VEGF) were associated with radiographic features of OA (FDR < 0.05). These same five biomarkers plus MCP-1 were statistically significantly associated with OA symptoms (FDR < 0.05) (Table 2). Interestingly, MMP-3 was associated with JSN severity while sVCAM-1 was associated with both JSN and OST severity. Although SF VEGF was associated with both structural and symptom severity of OA (KL, JSN, OST, and OA symptom scores), it was not associated with inflammation as reflected by etarfolatide scores. MCP-1 was associated only with symptom severity.

**Table 2:** Synovial fluid biomarkers associated with features of osteoarthritis (inflammation, radiographic, and symptom severity)

	Etarfolatide imaging	Radiographic severity			Symptom severity
	Synovial inflammation	KL	JSN	OST	PAS
Biomarker	Est. ×10^−2^ (95% CI) (pval)	Est. ×10^−2^ (95% CI) (pval)	Est. ×10^−2^ (95% CI) (pval)	Est. ×10^−2^ (95% CI) (pval)	Est. ×10^−2^ (95% CI) (pval)
sVCAM-1	0.970 (0.330–1.610) *(0.0029)*	0.160 (0.094–0.220) *(< 0.0001)*	0.130 (0.052–0.002) *(0.0009)*	0.430 (0.170–0.680) *(0.0010)*	0.320 (0.120–0.510) *(0.0015)*
MMP-3	52.712 (34.933–70.492) *(< 0.0001)*	9.591 (5.289–13.982*)* *(< 0.0001)*	6.477 (2.962–9.994) *(0.0003)*	17.695 (− 4.989–40.378) (0.1263)	15.220 (7.567–22.872) *(< 0.0001)*
sICAM-1	16.120 (3.020–29.220) (0.0159)	2.520 (1.400–3.630) *(< 0.0001)*	2.270 (0.970–3.570) *(0.0006)*	6.510 (3.250–9.780) *(< 0.0001)*	5.980 (2.480–9.480) *(0.0008)*
TIMP-1	1.470 (0.200–2.740) (0.0228)	0.200 (0.110–0.300) *(< 0.0001)*	0.140 (0.070–0.220) *(0.0002)*	0.610 (0.430–0.800) *(< 0.0001)*	0.790 (0.400–1.190) *(< 0.0001)*
VEGF	0.076 (− 0.310–0.460) (0.6985)	0.100 (0.047–0.140) *(0.0001)*	0.070 (0.013–0.130) *(0.0160)*	0.250 (0.120–0.370) *(0.0001)*	0.210 (0.130–0.300) *(< 0.0001)*
MCP-1	0.470 (− 0.670–1.610) (0.4159)	0.044(− 0.130–0.220) (0.6207)	0.036 (− 0.120–0.190) (0.6529)	0.310 (− 0.210–0.820) (0.2410)	0.420 (0.073 – 0.760) *(0.0175)*

Estimates (Est × 10^−2^) of regression coefficient (Beta estimate), 95% confidence intervals, and raw *p* values are listed in the table; those meeting FDR < 0.05 are italicized

*Abbreviations*: *KL* Kellgren-Lawrence; *JSN* joint space narrowing; *OST* osteophyte; *PAS* pain, aching and stiffness of knee; *pval p* value; *CI* confidence interval; *sVCAM-1* soluble vascular cell adhesion molecule 1; *MMP-3* matrix metalloproteinase-3; *sICAM-1* soluble intracellular adhesion molecule 1; *TIMP-1* tissue inhibitor of metallopeptidase inhibitor 1; *VEGF* vascular endothelial growth factor; *MCP-1* monocyte chemoattractant protein 1

### Correlations of synovial fluid biomarker concentrations with macrophage and neutrophil specific biomarkers

SF sVCAM-1, MMP-3, sICAM-1, TIMP-1, VEGF, and MCP-1 all correlated with markers specific for neutrophils (SF neutrophil elastase) and/or macrophages (SF CD14 and SF CD163) previously shown to predict knee OA progression [8, 16] (Table 3). These results suggest that this six-member panel of biomarkers reflects both macrophage- and neutrophil-mediated inflammation.

**Table 3:** Correlations of synovial fluid RBM biomarkers with known osteoarthritis progression biomarkers

OA Progression Biomarkers	Subset of synovial fluid RBM biomarkers associated with features of knee osteoarthritis*					
	sVCAM-1 *r* (95% CI) (pval)	MMP-3 *r* (95% CI) (pval)	sICAM-1 *r* (95% CI) (pval)	TIMP-1 *r* (95% CI) (pval)	VEGF *r* (95% CI) (pval)	MCP-1 *r* (95% CI) (pval)
CD14	0.86 (0.69–0.94) *(< 0.0001)*	0.77 (0.50–0.91) *(< 0.0001)*	0.84 (0.64–0.94) *(< 0.0001)*	0.88 (0.72–0.95) *(< 0.0001)*	0.90 (0.76–0.96) *(< 0.0001)*	0.50 (0.08–0.76) *(0.016)*
CD163	0.80 (0.56–0.92) *(< 0.0001)*	0.78 (0.51–0.91) *(< 0.0001)*	0.70 (0.37–0.88) *(0.0004)*	0.85 (0.66–0.94) *(< 0.0001)*	0.75 (0.44–0.90) *(0.0001)*	0.43 (− 0.02–0.73) *(0.046)*
Neutrophil Elastase	0.88 (0.68–0.96) *(< 0.0001)*	0.88 (0.68–0.96) *(< 0.0001)*	0.87 (0.64–0.96) (*< 0.0001*)	0.89 (0.71–0.97) *(< 0.0001)*	0.62 (0.16–0.86) *(0.008)*	0.14 (− 0.40–0.61) (0.581)

*As shown in Table 2; italicized *p* values pass FDR < 0.05; *r* = repeated measures correlation coefficient by Bland and Altman method implemented in rmcorr

*Abbreviations*: *RBM* Rules Based Medicine, *pval p* value, *CI* confidence interval, *sVCAM-1* soluble vascular cell adhesion molecule 1, *MMP-3* matrix metalloproteinase-3, *sICAM-1* soluble intracellular adhesion molecule 1, *TIMP-1* tissue inhibitor of metallopeptidase inhibitor 1, *VEGF* vascular endothelial growth factor, *MCP-1* monocyte chemoattractant protein 1

### Assessment of potential origin of biomarkers

Although plasma biomarker concentrations were not associated with any OA severity outcome, concentrations of one plasma biomarker, VEGF, correlated with its corresponding SF concentrations (Spearman’s rho *p* value ≤ 0.05, Additional file 1). Interestingly, plasma concentrations of VEGF also correlated with SF concentrations (averaged across both knees) of the other five inflammation associated biomarkers (sICAM-1, MCP-1, MMP-3, TIMP-1, and sVCAM-1). In addition, plasma MMP-3 correlated with SF concentrations of sVCAM-1. SF concentrations of VEGF, MMP-3, and TIMP-1 were all statistically significantly higher than the corresponding plasma concentrations (Additional file 2). Taken together, these results suggest that plasma VEGF may originate from the joint and reflect SF concentrations.

### Interactions of biomarkers associated with severity of OA inflammation, structural features, and symptoms

**Fig. 1:**
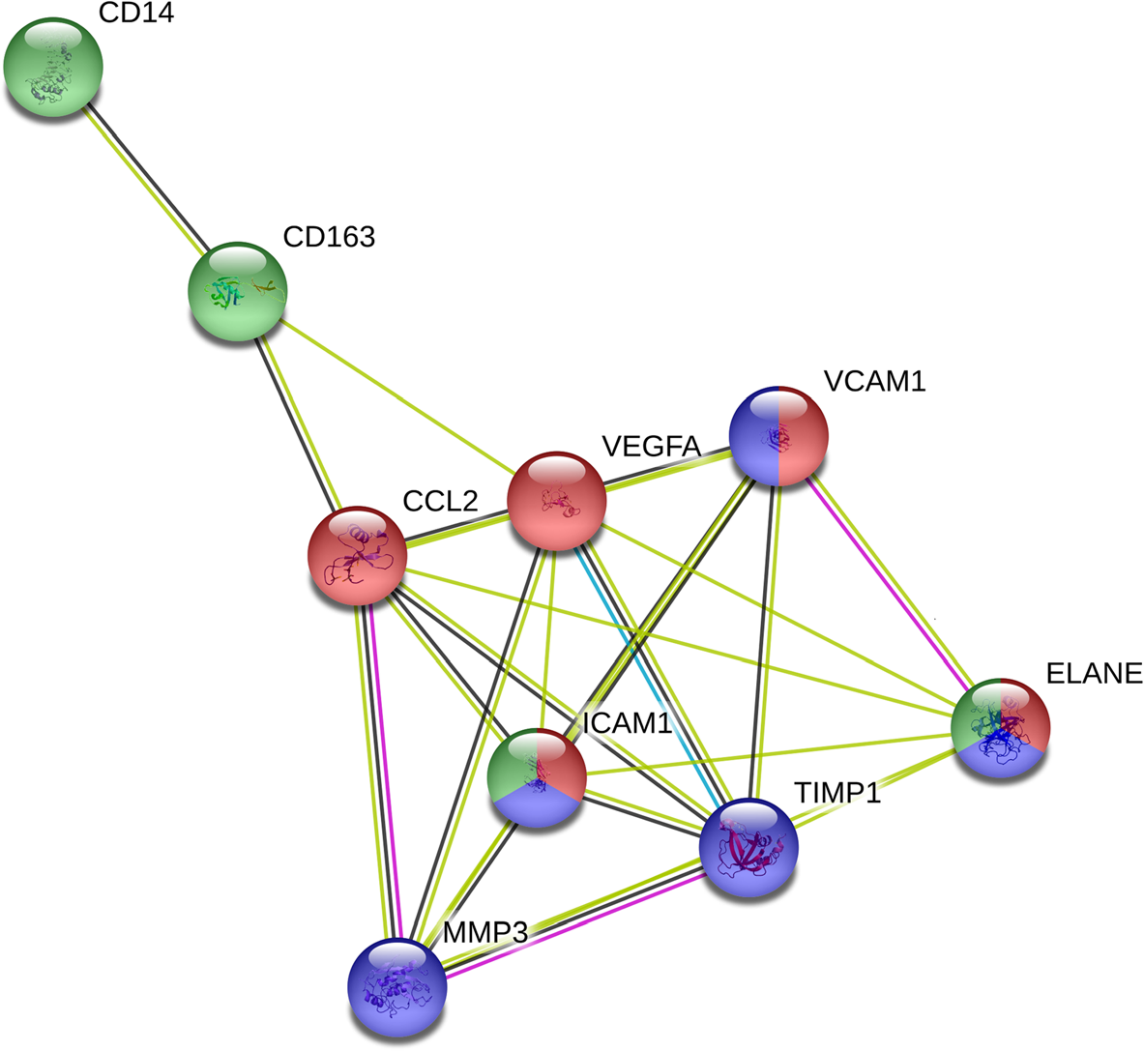
STRING plot showing functional biomarker interrelationships. Depicted relationships represent biomarkers with significant associations with OA inflammation, radiographic OA, and OA symptoms analyzed using the STRING v10.5 database. The colors of the spheres correspond to the biological processes in which a particular biomarker is involved. Sphere color key: leukocyte migration (red), extracellular matrix organization (blue), and inflammatory response (green). Joining string color key (corresponding to origin of data used for STRING database): curated databases (blue), experimentally determined (pink), textmining (yellow), and co-expression (black). Abbreviations: CD, cluster of differentiation 14; CD163, cluster of differentiation 163; CCL2 (also referred to as MCP-1 or monocyte chemoattractant protein 1), C-C chemokine motif 2 vascular cell adhesion molecule 1; ELANE, neutrophil elastase; ICAM-1, intracellular adhesion molecule 1; MMP-3, matrix metalloproteinase-3; TIMP-1, tissue inhibitor of metallopeptidase inhibitor 1; VEGF, vascular endothelial growth factor

We utilized STRING [26] to generate a functional protein association network for the six SF biomarkers (MMP-3, sVCAM-1, sICAM-1, VEGF, TIMP-1, and MCP-1) in this study with significant associations with etarfolatide (inflammation), KL (radiographic severity), and OA symptom scores. We added SF CD14, SF CD163, and SF neutrophil elastase to the model as biomarker surrogates for inflammation as we previously showed them to be strongly associated with the presence of folate receptor-positive cells in knee joints using etarfolatide imaging, strongly predictive of knee OA progression [8, 16], and correlated with the OA-related RBM markers of the final panel. Relevant biological pathways strongly associated with these nine biomarkers include leukocyte migration and extracellular matrix organization (Fig. 1). This plot is consistent with these biomarkers functioning both as extracellular signaling molecules and as effectors of OA progression.

## Discussion

**Fig. 2:**
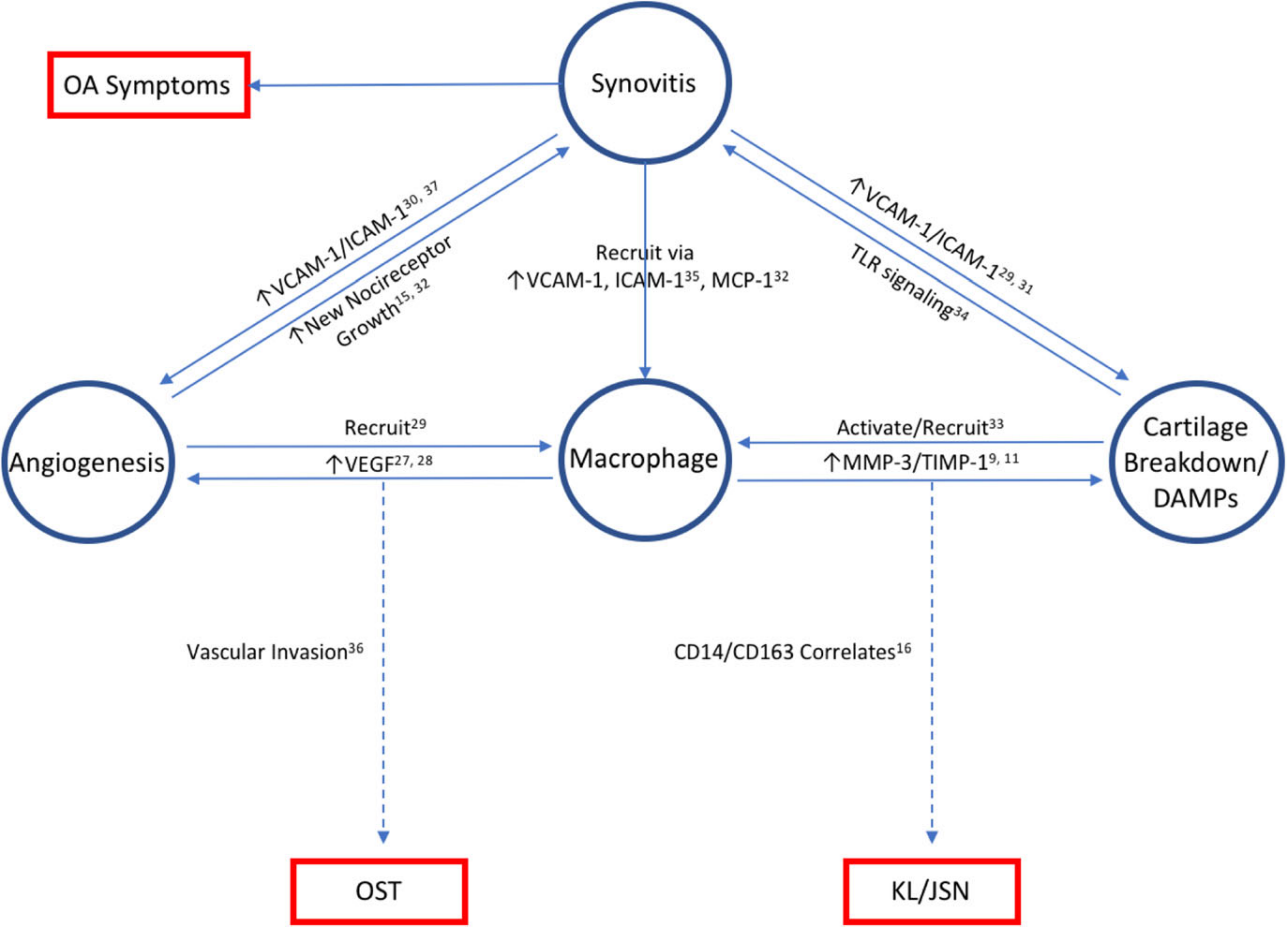
Inflammatory OA endotype based on synovial fluid biomarkers. This plot conceptualizes our data in the context of the current understanding of inflammation in OA. Abbreviations: OA, osteoarthritis; DAMPs, disease-associated molecular patterns; VCAM-1, vascular cell adhesion molecule 1; MMP-3, matrix metalloproteinase-3; ICAM-1, intracellular adhesion molecule 1; TLR, toll-like receptor; TIMP-1, tissue inhibitor of metallopeptidase inhibitor 1; VEGF, vascular endothelial growth factor; MCP-1, monocyte chemoattractant protein 1; KL, Kellgren-Lawrence; JSN, joint space narrowing; OST, osteophyte; NHANES, national health and nutrition examination survey measure of OA symptoms (pain, aching, stiffness)

MMP-3, sVCAM-1, sICAM-1, VEGF, TIMP-1, and MCP-1 were all associated with synovial inflammation by etarfolatide imaging, radiographic OA severity, and/or OA symptoms. Although soluble VCAM-1, sICAM-1, and VEGF have no known intrinsic ability to cause cartilage loss, they were strongly associated with severity of cartilage loss based on JSN and macrophage and neutrophil markers (SF CD14, SF CD163, and SF elastase) that we found previously to be associated with OA progression [8, 16]. Etarfolatide imaging, indicating the presence of activated macrophages and neutrophils in the synovial tissue [8], provided additional in vivo evidence for synovial inflammation that was positively associated with SF biomarkers MMP-3 and sVCAM-1. These results are consistent with the inflammation and arthritis promoting roles of these molecules (summarized in Fig. 2) [9, 11, 15, 16, 27–37]. In particular, VCAM-1 is expressed by all cell types of the joint organ, chondrocytes, synovial fibroblasts, and adipocytes. Because VCAM-1 binds leukocytes [35], soluble VCAM-1 serves as a chemotactic stimulus for macrophages [36], and DAMPS and MCP-1 serve as chemotactic stimuli for macrophages and neutrophils [38], thereby providing direct means for macrophages and neutrophils to home to the joint to promote cartilage degradation.

Of the six biomarkers, MMP-3, VEGF, and TIMP-1 were more abundant in SF than plasma supporting a joint tissue origin. The finding that SF, but not plasma biomarkers, correlated with OA severity outcomes, taken together with existing literature on these analytes in OA, further supports the concept that these markers are specific to local disease-related phenomena in the joint organ [39]. For instance, MMP-3, a well-known collagen degrading protease associated with OA progression [29], is known to contribute to cartilage breakdown with the generation of DAMPs [40, 41]. These DAMPs can directly activate macrophages and neutrophils [33] and increase synovial inflammation through toll-like receptor signaling [34, 40]. VEGF is a signal protein of angiogenesis that can increase inflammation through creation of blood vessels capable of leukocyte recruitment [27, 28]. VEGF injected into the temporomandibular joint increased MMP-9, MMP-13, the initiation of OA, and cartilage loss [42]. VEGF inhibition, in surgically induced (destabilization of medial meniscus) OA, reduced OA progression [43]. VEGF also acts as a chemoattractant and activator of macrophages [44]. Once macrophages are recruited to the joint, they have the ability to produce more VEGF and promote further angiogenesis, symptoms [45], and synovitis that will facilitate macrophage recruitment and perpetuate joint inflammation [29].

Although the hallmarks of osteoarthritis, OA pain, aching, and stiffness, are highly heterogeneous, a major contributor to OA symptoms is synovitis [29]. It is therefore understandable that synovial fluid biomarkers shown here (sVCAM-1, MMP-3, sICAM-1, VEGF, TIMP-1) and previously (CD14) to be associated with synovial inflammation (as reflected by etarfolatide imaging scores) [16] were also associated with joint pain, aching, and stiffness. These markers may therefore have value as indicators of a symptomatic inflammatory OA endotype. These results are in agreement with previous studies showing that sVCAM-1 and sICAM-1 are associated with synovitis [36, 46]. Moreover, VEGF can also lead to cartilage vascularization at sites that subsequently form osteophytes [47]; osteophytes are a feature of OA known to contribute to symptoms [15].

Limitations of this study include the small cohort size, especially in comparison to the number of outcomes measured. However, to mitigate this limitation, multiple measures correction was performed by adjusting *p* values using the Benjamini-Hochberg false discovery rate [24] method. Due to the cross-sectional analysis and absence of longitudinal data, causality of biomarkers with OA progression could not be inferred. Although we do not have longitudinal data, several of these biomarkers have face validity for involvement in OA progression based on the literature including VEGF, ICAM1, VCAM1, and MMP3 [6, 31, 48–51]. When direct aspiration of SF failed, low volume saline lavage was performed. Using both direct and lavage SF in this study is both a strength and a limitation. On the one hand, this is a strategy that allows potential evaluation of the highest number of knees, even less inflamed ones without appreciable SF; on the other hand, the knees requiring lavage tend to be less inflamed, having lower inflammatory biomarker concentrations that are even lower with lavage. As expected, more biomarker concentrations from lavage samples were below lower limits of detection of the inflammation map multiplex assays. Nevertheless, we were partially able to overcome these challenges with biomarker selection criteria, the well-established urea correction method [23] and a well-established imputation methodology. Although only 17 of 47 SF biomarkers passed our inclusion criteria for statistical analysis, the biological credibility of the results attests to the viability of this strategy.

## Conclusions

In summary, this study provides support for an inflammatory knee OA endotype characterized by the presence in the synovium of activated macrophages and neutrophils and their association with SF biomarkers of inflammation and angiogenesis. These data support a highly integrated process with VEGF, a biomarker of angiogenesis [37], increasing expression of MMPs and inflammation, based on markers of vascular adhesion, sVCAM-1 and sICAM-1, correlated with synovitis [36]. This biomarker profile may assist in identifying a specific subgroup of patients for treatments modulating inflammation and activated macrophages and neutrophils.

## Additional files

Additional file 1: Correlation of biomarkers in plasma with synovial fluid. SF biomarkers were correlated to corresponding plasma biomarkers using Spearman correlation. (DOCX 16 kb)

Additional file 2: Wilcoxon signed ranks for SF vs. plasma. Wilcoxon signed-rank statistic shows whether the biomarker was higher in SF or plasma. (DOCX 13 kb)

## Abbreviations

A2M: Alpha-2-Macroglobulin
AAT: Alpha-1-Antitrypsin
B2M: Beta-2-Microglobulin
C3: Complement C3
CV: Coefficient of Variation
DAMPs: Damage-associated molecule patterns
FRTN: Ferritin
GEE: Generalized estimating equations
ICAM-1: Intercellular adhesion molecule 1
IL-1ra: Interleukin-1 receptor antagonist
IQR: Interquartile range
JSN: Joint space narrowing
KL: Kellgren-Lawrence
LLOD: Lower limit of detection
MCP-1: Monocyte chemotactic protein 1
MMP: Matrix metalloproteinase
MMP-3: Matrix metalloproteinase-3
MMP-9: Matrix metalloproteinase-9
OA: Osteoarthritis
OST: Osteophyte
PARs: Proteinase-activated receptors
SCF: Stem cell factor
SF: Synovial fluid
TIMP-1: Tissue inhibitor of metalloproteinases 1
ULOD: Upper limit of detection
VCAM-1: Vascular cell adhesion molecule-1
VDBP: Vitamin D-binding protein
VEGF: Vascular endothelial growth factor
