# Supplementary material for: Synovial fluid biomarkers associated with osteoarthritis severity reflect macrophage and neutrophil related inflammation
Source: Arthritis Res Ther. 2019 Jun 13;21:146. doi: 10.1186/s13075-019-1923-x (PMC6567574; doi:10.1186/s13075-019-1923-x)
Supplement: Supplementary file 1 — Correlation of biomarkers in plasma with synovial fluid. SF biomarkers were correlated to corresponding plasma biomarkers using Spearman correlation. (DOCX 16 kb) [file 13075_2019_1923_MOESM1_ESM.docx]

**Additional file 1. Correlation of biomarkers in plasma with synovial fluid**

|  |  | Plasma | | | | | |
| --- | --- | --- | --- | --- | --- | --- | --- |
|  | r  (pval) | sICAM-1 | MCP-1 | MMP-3 | TIMP-1 | sVCAM-1 | VEGF |
| Synovial fluid | sICAM-1 | 0.171  (0.413) | -0.195  (0.349) | 0.282  (0.173) | 0.247  (0.234) | 0.394  (0.051) | **0.542**  **(0.005)** |
|  | MCP-1 | 0.140  (0.504) | 0.292  (0.157) | -0.280  (0.176) | 0.367  (0.071) | 0.332  (0.105) | 0.390  (0.054) |
|  | MMP-3 | 0.043  (0.837) | -0.270  (0.191) | 0.297  (0.150) | 0.200  (0.339) | 0.337  (0.100) | **0.396**  **(0.050)** |
|  | TIMP-1 | -0.076  (0.719) | -0.345  (0.091) | 0.363  (0.075) | 0.113  (0.590) | 0.273  (0.187) | **0.442**  **(0.027)** |
|  | sVCAM-1 | 0.011  (0.958) | -0.288  (0.162) | **0.407**  **(0.043)** | 0.172  (0.410) | 0.318  (0.121) | **0.522**  **(0.007)** |
|  | VEGF | 0.170  (0.415) | -0.284  (0.169) | 0.326  (0.112) | 0.215  (0.301) | 0.308  (0.134) | **0.614**  **(0.001)** |

SF biomarkers were correlated to corresponding plasma biomarkers using Spearman correlation. Values in bold represent significant rho with p values ≤ 0.05.

Abbreviations: r: Spearman’s rho; pval: p value; ICAM-1: intercellular adhesion molecule 1; MCP-1: monocyte chemotactic protein 1; MMP-3: matrix metalloproteinase-3; TIMP-1: tissue inhibitor of metalloproteinases 1; VCAM-1: vascular cell adhesion molecule-1; VEGF: vascular endothelial growth factor.
