# Supplementary material for: Synovial fluid biomarkers associated with osteoarthritis severity reflect macrophage and neutrophil related inflammation
Source: Arthritis Res Ther. 2019 Jun 13;21:146. doi: 10.1186/s13075-019-1923-x (PMC6567574; doi:10.1186/s13075-019-1923-x)
Supplement: Supplementary file 2 — Wilcoxon signed ranks for SF vs. plasma. Wilcoxon signed-rank statistic shows whether the biomarker was higher in SF or plasma. (DOCX 13 kb) [file 13075_2019_1923_MOESM2_ESM.docx]

| SF vs Plasma | Test Statistic S | P value |
| --- | --- | --- |
| MMP-3 | 162.5 | **2.62338E-09** |
| VEGF | 156.5 | **3.43555E-08** |
| TIMP-1 | 148.5 | **4.90062E-07** |
| sICAM-1 | -162.5 | **2.62338E-09** |
| MCP-1 | -151.5 | **1.95635E-07** |
| sVCAM-1 | -47.5 | 0.103771301 |

**Additional file 2. Wilcoxon signed ranks for SF vs. plasma**

Sign of S, denoting Wilcoxon signed-rank statistic, shows whether the biomarker was higher in SF (positive) or plasma (negative). Bolded values signify p value ≤ 0.05.

Abbreviations: SF: synovial fluid; S: Wilcoxon signed-rank statistic; pval: p value; ICAM-1: intercellular adhesion molecule 1; MCP-1: monocyte chemotactic protein 1; MMP-3: matrix metalloproteinase-3; TIMP-1: tissue inhibitor of metalloproteinases 1; VCAM-1: vascular cell adhesion molecule-1; VEGF: vascular endothelial growth factor.
